# Reviews

**Published:** 1923-04

**Authors:** 


### REVIEWS OF BOOKS.

### RECOLLECTIONS OF A GREAT SURGEON.

**The Elephant Man and other Reminiscences.** By Sir FREDERICK TREVES. (Cassell; 7s. 6d. net.)

To priest and doctor are confided the most intimate secrets of humanity, and it is as studies in psychology that these sketches make their chief appeal. But such a chapter as "The Old Receiving Room," with the lurid light it throws upon past methods, is of exceptional interest, and may even help to persuade Mr. Bernard Shaw that there is something to be said for modern aseptic surgery:—

"There was no object in being clean. Indeed cleanliness was out of place. It was considered to be finicking and affected. An executioner might as well manicure his nails before chopping off a head. The surgeon operated in a slaughter-house-suggesting frock-coat of black cloth. It was stiff with the blood and the filth of years. The more sodden it was the more forcibly did it bear evidence to the surgeon's prowess. I, of course, commenced my surgical career in such a coat, of which I was quite proud. Wounds were dressed with 'charpe' soaked in oil. Both oil and dressing were frankly and exultingly septic—'charpe' was a species of cotton waste obtained from cast linen. It would probably now be discarded by a motor mechanic as being too dirty for use on a car. Owing to the suppurating wounds the stench in the wards was of a kind not easily forgotten."

Indeed, Sir Frederick remembers a whole ward being decimated by hospital gangrene. "People," he adds, "often say how wonderful it was that surgical patients lived in those days. As a matter of fact they did not live, or at least only a few of them. Lord Roberts assured us that on the Ridge at Delhi during the Indian Mutiny no case of amputation recovered. This is an extreme instance, for the conditions under which the surgeons on the Ridge operated were exceptional and hopelessly unfavourable."

The story of "the Elephant Man" is one of peculiar pathos. This dreadful deformity was discovered by Sir Frederick in the early eighties when he was being exhibited by a showman in the Mile End Road. Too terrible in face to go out unmasked, crippled and with distorted limbs, apparently imbecile, this poor creature had never known anything but harsh treatment and isolation from all other human beings. Sir Frederick rescued him from this appalling life, and he found sanctuary in two rooms at the London Hospital. There, expanding under care and kindness, he was found to be possessed of acute intelligence, and of a gentle and loveable disposition. He who had never been smiled at by a woman was visited by almost every lady of note in the social world:—

"The Merrick whom I had found shivering behind a rag of a curtain in an empty shop was now conversant with Duchesses and Countesses and other ladies of high degree. They brought him presents, made his room bright with ornaments and pictures and, what pleased him more than all, supplied him with books. He soon had a large library, and most of his day was spent in reading. . . . The height of his social development was reached on an eventful day when Queen Alexandra—then Princess of Wales—came to the hospital to pay him a special visit. With that kindness which has marked every act of her life, the Queen entered Merrick's room smiling and shook him warmly by the hand. Merrick was transported with delight."

Sir Frederick has not much to say of his own share in bringing about this transformation in the life of the poor "Elephant Man," but it is clear that he is responsible for most of the happiness it was possible for Merrick to enjoy. A grim little sketch is "The Idol with Feet of Clay"—the story of a young surgeon who attempted to operate on his wife for appendicitis. Immensely self-confident, he was readily induced by her to undertake the operation, anxious to show that here was a man "who could carry out a grave operation on his wife without a tremor." The operation seemed simple—in nine out of ten cases, points out Sir Frederick, it is simple; but this, alas! was the tenth case. In the trying circumstances the operation proved beyond him; his wife succumbed, and he felt himself to be her murderer. That the "ruling passion is strong in death" is illustrated by his story of the eminent journalist who "always wanted to know." "When he was *in extremis*, but still capable of recognising those around him, the dire sound of rattling in his throat commenced. He indicated that he wanted to speak to me. I went to his bedside. He said, in what little voice remained, 'Tell me, is that the death rattle?' I replied that it was. 'Thank you,' he said, with a faint shadow of a smile; 'I thought so.'"

This is one of those rare books in which there is not a dull line, and, in addition to the absorbing interest of the stories he has to relate, Sir Frederick tells them with a grace of expression which gives them real literary distinction.

### EATING AND DRINKING.

**The Diet of Women.** By CECIL WEBB-JOHNSON, M.B., Ch.B. (Mills & Boon; 5s. net.)

Diet is a subject of never-ending interest and discussion; hence the many books thereon. Dr. Webb-Johnson has published this one to enlighten his readers on what they need, and what they eat and drink to excess. It is written in pleasant phraseology, and is free from many technical terms. He states the proportion of protein and other constituents in various foods, and writes convincingly on the evils following excessive eating, especially of flesh-foods. In his opinion we all eat too much of everything; but we do not find a clear statement of the actual amount of protein, fat, and carbohydrate that we ought to eat in various circumstances, nor how to obtain them from various sources. He shows a bias towards vegetarianism, and declares that "nuts and fruit are the natural food of man." But the frequency of the sin of gluttony does not prove that everybody is a glutton, or that the average Briton eats too much meat. Many people who can afford to do so, eat more meat, poultry, fish and eggs than they really need; but these form a minor section of the nation. Dr. Webb-Johnson discusses favourably a "No-breakfast plan." Experience, however, shows that the average person needs three meals a day, and if the millions of people who work from three to five hours before noon followed the "No-breakfast plan" they would soon be unable to work at all. There is a small percentage of people who, from idiosyncrasy, are better without breakfast or with merely a cup of tea or coffee and a piece of toast. But these probably are descendants of a family who for generations had a heavy meal in the evening.

Whether one or three meals be eaten, the food must contain a due proportion of things necessary for the well-being of the body. Not less than $2\frac{1}{2}$ ounces of dry protein, 1 ounce of fat, and 12 ounces of carbohydrate, yielding 1,750 calories, are needed for a resting person; while Ranke found $3\frac{1}{2}$ ounces of dry protein, $3\frac{1}{2}$ ounces of fat, and $8\frac{1}{2}$ ounces of carbohydrate, yielding 2,310 calories, suitable for a professional man or other person following a light occupation. The first is the minimum necessary; the maximum is consumption to repletion. But there is also an *optimum* or quantity which is necessary for the individual according to his work. Trouble always follows unbalanced rations. It was formerly said, "Look after the calories and the protein will take care of itself," but the reverse is nearer the truth. The author's zeal against overfeeding leads him to make statements which will not bear strict analysis. He says, for instance, that "meat has little nutritive value," and that "poultry and game are neither so nutritious nor so wholesome." These statements are not borne out by a comparison of the chemical composition and heat-value of the foods mentioned. The higher calorie value of nuts and beans is due to their containing carbohydrates. There is no carbohydrate in meat, poultry and fish; hence we eat bread and potatoes with them to supply the deficiency. Experiment has proved that poultry is digested more easily and quickly than meat; hence the value of chicken for an invalid. It is true that some people enjoy better health on a vegetarian diet, but all do not. When well-carried out, vegetarianism is an expensive hobby, needing a first-class cook, and an unlimited supply of eggs and butter, which are not vegetables. Few people care to live on a diet of beans, nuts, fruit and vegetables, nor is it necessary that they should.

To nursing mothers, Dr. Webb-Johnson says, "Fresh fruit, green salads, and water make better milk than meat, fish, cream and stout." But science has proved that during pregnancy and nursing women need a larger ration of meat than in ordinary times; they require more protein, and they will not get it from fruit and vegetables. Milk has ever been found a good food for mothers; but we are here told that "milk is not a proper food for adults." The reasons he gives are inadequate. An adult can live for weeks on 4 pints of milk a day, yielding 1,600 calories, about enough for a person in bed. Most of what the author says against milk would, however, be useful in "The Clean Milk Campaign." There are bacteria in milk fresh from the cow, but they are those which turn it sour, and are quite innocent; they never hurt anybody. The presence in milk of germs which cause typhoid, diphtheria, &c., is quite accidental. Tubercle bacilli do not exist in the milk of healthy cows. Milk puddings do not meet with the author's approval, but his outcry against them will not find favour; the usefulness of milk puddings cannot be impugned. They are free from germs, easily digested, and suitable for all persons except diabetics.

The account Dr. Webb-Johnson gives of vitamins is useful and puts them in the right light; he says, however, that they are destroyed by canning, which is an unsettled point, although they are largely destroyed by boiling. Drinking at meals is prohibited by him, especially tea and soup. Science, however has proved that a few spoonsful of soup at the beginning of dinner are beneficial. He rightly speaks against excessive tea-drinking. It is an unnecessary stringency to forbid any liquids with a meal. Many people with poor appetite can eat more when they swallow a little water, beer or wine at various times between their mouthfuls of food. The error arises from wetting the food with our drink instead of with saliva. There are many excellent passages and much good advice drawn from experience in the book, although we are unable to accept all the author's views.

### DOCTOR AND EDUCATIONIST.

**Life and Work of Sir James Kay-Shuttleworth.** By FRANK SMITH. (John Murray; 18s. net.)

The profession of medicine has the distinction of having trained many men who have also become famous in other fields, from the present Poet Laureate to, for instance, the subject of this memoir, who, Mathew Arnold declared, deserved a statue for his share in the making of elementary education in England. To him principally is due the presence of a school in every area, the existence of the Board of Education, and, by derivation, every educational provision by the public authorities that we now take as a matter of course. But all was won despite the apathy, indifference and opposition that attend upon every revolutionary change, however peaceful. The bulk of this book, in which an extraordinary mass of information has been skilfully compressed, necessarily deals with the culminating years at the Education Office, and though the story is of interest to every intelligent reader, and is excellently told, we must here confine ourselves to the consideration of how a young doctor converted himself into an educational pioneer of this high order.

The son of a Lancashire cotton manufacturer, James Kay, who took his second surname upon his marriage, was a child of the industrial revolution. Born in 1804, and inheriting his parents' religious zeal, he was brought up at Salford and Rochdale, and at fifteen joined his uncle, a Rochdale banker. There he became an active Sunday-school teacher, till he left in 1824 to become a medical student at Edinburgh University, where he quickly made his mark, not only as a student, but as a debater. He interested himself in research, and collected his results into the causes of death by asphyxia, which, in a later form, have survived to receive the praise of Sir James Mackenzie. It was, however, as medical assistant in the Edinburgh New Town Dispensary that he began to find his vocation. His visits made him familiar with slum life in its most atrocious aspects, and he became early absorbed in the relation of social conditions to disease, and saw that the one could not be remedied without improving the other.

This led him to study political economy, and on taking his degree in 1827 he began to practise in Manchester. The evils that he met there among the slums, with no arterial drainage, and no proper water supply, turned his attention to the Reform Bill, then being agitated. The cholera outbreak of 1832 completed an indelible impression, and led to his publication of a pamphlet in which his exposure of industrialism was accompanied by a demand, among other things, for education.

His views, based on personal observation and supported by statistics, attracted the attention of the Government, and at the moment when his professional prospects and his health were imperilled by his sanitary agitation, he was offered, and accepted, an assistant Poor Law Commissionership in East Anglia. This was the turning point of his life, for the conditions that the new Poor Law was intended to relieve convinced him that if the workhouse child was to become a self-supporting citizen it must be properly taught and be made an asset to employers by its developed intelligence and education. To do this teachers had to be found and trained, and likely pupils promoted to the duties of pupil teachers. The immense task, developing finally into the Education Act of 1870, filled the life which Mr. Frank Smith has composed into a fascinating narrative. Shuttleworth's combination of scientific method with philanthropic motive was his great quality,and this book does tardy but careful justice to his work. As Mathew Arnold also said, it needed a statesman to appreciate him; and, as we have seen, even in medicine he is still rememberable. In him the born administrator and the great task were simultaneously found, to the lasting benefit of the nation. There is not a dull page in this important contribution to educational history.

### HOW SOON DOES CONSUMPTION KILL ?

**A Contribution to our Knowledge of the Clinical Course and Duration of Fatal Lung Tuberculosis.** By Dr. C. H. Würtzen, Copenhagen. (Gyldendal, Hanover Square, W.; 2s. 6d. net.)

Many have been the attempts to estimate the average duration of life in pulmonary tuberculosis, but there has always been difficulty in fixing the starting-point for the disease with anything like certainty. From three to seven years has been given as an average by various authorities. Something approaching to accuracy may be obtained by dealing with fatal cases and by taking the beginning of the disease as the date of appearance of the first symptom. On this basis—and not that of the date of notification—Dr. Würtzen made investigations over a period of nearly fourteen years at the Tuberculosis Department of the Oeresund Hospital, Copenhagen. The cases comprised 1,032 men, 780 women and 87 children. He found that the mean duration for adults was 35·9 months—34·4 months for women and 37·1 months for men. The greater fatality among children is shown by his figure of 11·9 months. When pulmonary tuberculosis was associated with diabetes mellitus, the mean duration was 7½ months, and when combined with syphilis 27·5 months. The younger the patient the less was his resistance to the disease. These results should be helpful in prognosis as to the expected duration of life, though, of course, they do not concern the large number of cases of arrest of the disease.

### THE PROBATIONER NURSE.

**Hints to Probationer Nurses in Mental Hospitals: with a brief Introduction to Psychology.** By Richard Eager, O.B.E., M.D. (Aberd.), Neurologist, the Ashurst Special Neurological Hospital, Oxford, etc. (H. K. Lewis & Co., Ltd.; 1s. 6d. net.)

This little book fills a distinct gap in nursing literature, probationers in mental work being not nearly so abundantly provided for as the beginner in a general hospital. Entirely of an elementary character, the book is intended exclusively for those who are starting their duties in mental wards without any previous training in such work. It begins with the reasons for the standing orders obtaining in all mental hospitals, and thus explains what at first must seem very arbitrary and irksome rules. A short chapter follows on a few of the principal emergencies likely to occur among those of unsound mind, with hints on how to act in such cases. Fits, choking, attempts at suicide and alarms of fire are the most frequent. Some general advice on various ward duties is given in a short chapter, together with useful hints on the necessary procedure for self-defence in the case of a homicidal patient becoming violent. Rudimentary principles on which may be based the proper carrying out of ward duties only are dealt with in the book, but they embrace all essentials of intelligent treatment of the mentally afflicted. The rest of the book is a short introduction to the study of psychology—a difficult though fascinating subject, which the author makes wholly interesting.

---

### MISCELLANEOUS NOTICES.

### The Medical and Dental "Who's Who."

The Medical Register, 1923; The Dentists' Register, 1923; Minutes of the Dental Board, Vol. I., 1922; List of Medical and Dental Students Registered During the Year 1922. (Constable, for the General Medical Council.)—The "Medical Register" is the one indispensable book of reference not only to the medical man, but to everyone who is practically concerned with the public health. As an official publication its authority is complete, and its accuracy beyond question, and it is brought up to the latest possible moment. The annual revision of so vast a mass of names, addresses, dates and qualifications is a complicated and meticulous task which has once more been performed with complete efficiency. But the "Register" is something more than a list of qualified practitioners—it is the readiest means of reference for the crucial medical and dental legislation of the last sixty years or more. The "Dentists' Register" is equally valuable and authoritative. The list of students also has its uses, while it is only the foolish virgins among the dentists who will fail to furnish themselves with the "Minutes of the Dental Board."

### For the Student.

Surgical Tables. Obstetric Tables. By Maurice C. Anderson, L.R.C.P. A. & C. Black. 3s. 6d. net each.—Second editions of two handy little books, primarily intended for the medical student but, as their author hopes, likely also "to be of some use to the young practitioner." Both books has been thoroughly revised and brought up to date, and in "Obstetric Tables" certain important sections have been entirely re-written. The matter is clearly set out and well arranged, and there is an excellent index.

